# Thermal Transport in a 2D Nanophononic Solid: Role of bi-Phasic Materials Properties on Acoustic Attenuation and Thermal Diffusivity

**DOI:** 10.3390/nano9101471

**Published:** 2019-10-16

**Authors:** Haoming Luo, Anthony Gravouil, Valentina Giordano, Anne Tanguy

**Affiliations:** 1 LaMCos, INSA-Lyon, CNRS UMR5259, Université de Lyon, F-69621 Villeurbanne Cedex, France; Haoming.Luo@insa-lyon.fr (H.L.); Anthony.Gravouil@insa-lyon.fr (A.G.); 2 Institut Lumière Matière, UMR 5306 Université Lyon 1-CNRS, F-69622 Villeurbanne Cedex, France; Valentina.Giordano@univ-lyon1.fr

**Keywords:** acoustic and thermal transfer, nanocomposite material, nanophononic material, numerical simulations, 62.20.-x

## Abstract

Nanophononic materials have recently arisen as a promising way for controlling heat transport, mirroring the results in macroscopic phononic materials for sound transmission, filtering and attenuation applications. Here we present a Finite Element numerical simulation of the transient propagation of an acoustic Wave-Packet in a 2D nanophononic material, which allows to identify the effect of the nanostructuration on the acoustic attenuation length and thus on the transport regime for the vibrational energy. Assuming elastic behavior in the matrix and in the inclusions, we find that the rigidity contrast between them not only tunes the apparent attenuation length of the wave packet along its main trajectory, but gives rise to different behaviours, from weak to strong scattering, and waves pinning. As a consequence, different energy transport regimes can be identified in the three-parameter space of the excitation frequency, inclusions size and rigidity contrast, leading to the identification of a combination of parameters allowing for the shortest attenuation distance. These results could have applications both in the field of acoustic insulation, and for the control of heat transfer.

## 1. Introduction

In the last years technological developments have known an important slowing down, mainly due to the need of dealing with the heat problem [1]: for example, the extreme miniaturization of electronic devices inevitably leads to a local overheating and dissipation (in the central processing unit for example) which is detrimental to the device performance and lifetime. In order to allow for a further miniaturization, it is fundamental to be able to drive away the extra-heat, and thus novel materials with an enhanced thermal conductivity are sought. On the other hand, the omnipresence of heat in the use of technology calls for an efficient way of recycling it and transforming it into a usable source of energy. This can be achieved through the thermoelectric conversion, whose efficiency depends on the capability of materials of keeping a temperature gradient constant, and thus on their good thermal insulation. As such, the development of means for controlling heat transfer, its propagation direction and efficiency, is at the heart of the current research in thermal science as well as microtechnology [2,3,4,5,6,7,8,9,10].

**Figure 1:**
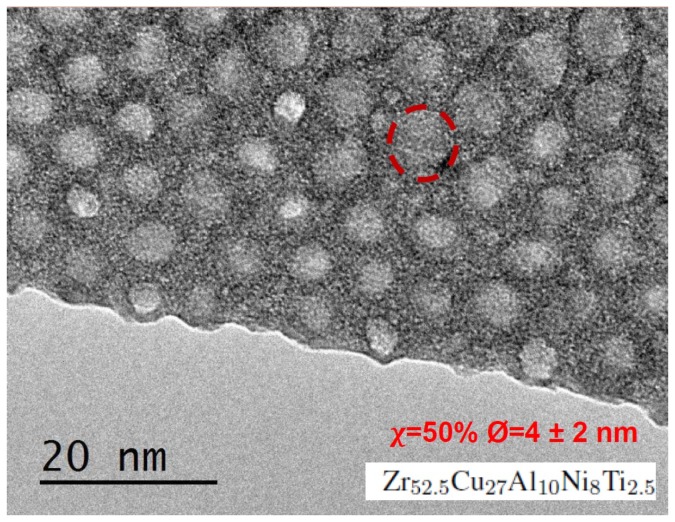
Transmission Electron Microscope image of a Zr-Cu-Al-Ti-Ni metallic glass with nano- crystalline inclusions, taken from [27]

Heat transfer is actually intimately related to the sound propagation (acoustic transfer) in materials, as in insulators and semi-conductors the main heat carriers are acoustic phonons [11]. As such, the specific dynamics of phonons will directly affect thermal transport, with two different contributions: a propagative one, which depends on phonon mean-free path, heat capacity, velocity and vibrational density of states [12], and a diffusive one, involving the phonon diffusivity rather than the mean-free path and velocity [13,14,15]. The propagative contribution can be reduced through the presence of interfaces, which scatter phonons. This concept has indeed been largely exploited for efficiently manipulating long-wavelength acoustic phonons, which assure sound propagation at low frequency, through the introduction in materials of periodic interfaces on a macroscopic scale (phononic crystal) [10,16,17,18]. Depending on the actual design and the contrast of properties between the materials on the two sides of the interface, acoustic filters or guides have successfully been built [6,19,20,21,22]. More recently, the same concept has been proposed for manipulating phonons with nanometric and sub-nanometric wavelengths and frequencies in the THz regime, which are the dominant heat carriers at room temperature. On the experimental side, nanocomposites made of the intertwining of two phases in a continuous network or in the form of a major phase, the matrix, with nanometric inclusions of a secondary phase embedded in (Figure 1), have been largely investigated, clearly showing that the effect on thermal transport dramatically depends on the properties of the two parent phases and the detailed nanostructure. While phonon scattering in systems with interfaces is very well known in some limiting cases (low interface density, low or large rigidity contrast in the scatterers ... [23]), it can be more complex in intermediate regimes, especially when the volume fraction of inclusions exceeds few tens of percents. Moreover, out-of-equilibrium thermal transport is related to transient dynamics of phonons, and not to stationary properties. And indeed, both thermal conductivity reduction and enhancement have been reported in different systems [24,25,26], and even a disappointing lack of effect has been evidenced in metallic glasses with nano-crystalline inclusions, likely due to the very similar electric and phonon properties between the two phases [27]. Many theoretical studies have tried to shed light onto these very different behaviors and the key parameters determining the effect of the interfaces on thermal transport. If the majority of these have been limited to the calculation of the thermal conductivity, some works have looked at the phonon dynamics to get a better insight on transport properties and found exotic behaviors such as an energy localization between pores [28], or the filtering of high frequency phonons [20].

Recently, we have shown that interfaces affect differently phonons of different wavelengths and frequencies and it is fundamental to look at all phonons relevant for heat transport at a certain temperature, and their perturbed dynamics, for being able to understand thermal conductivity in such nanocomposites [7,29,30]. In that work we could highlight that the rigidity contrast between the two phases is a determinant parameter ruling the strength of the scattering which concerns the phonons with a wavelength comparable with the nanostructuration lengthscale. However, thermal conductivity will be affected only if those phonons are the most relevant for thermal transport. Understanding thermal transport in such materials requires then an extensive investigation of the combined effect of rigidity contrast, interface density and phonon wavelength, for mapping the multi-dimensional parameters space and identifying the optimum conditions for thermal conductivity reduction or enhancement.

This is the aim of this work, which proposes to perform with finite elements calculations a systematic parametric study of the attenuation of acoustic wave-packets through the transient dynamics of phonons, as a function of the excitation frequency, the inclusions volume fraction and elastic contrast. The attenuation length of the wave-packets along a given direction will allow quantifying the mechanical (and thermal) energy transfer in that direction and identify the transfer regime, from ballistic to diffusive to localized. This approach simultaneously describe two physical phenomena, different only for the lengthscale involved: the propagation of high frequency pulses in nanophononic materials (acoustic meta-materials) and the heat propagation in the same nanocomposites (thermally engineered nanomaterials). It is important to mention that heat propagation is assured, as said, by phonons, that is vibrations with well defined frequencies (waves) but finite coherence time (particles). In crystals, normal modes are phonons with infinite coherence time, but in amorphous solids like glasses, the normal modes are not plane waves [31]. As such, using a wavepacket with finite coherence time allows a description of the vibrations preventing the permanent mixing with other plane waves. Wavepackets are thus a good representation for phononic excitations in amorphous materials, and - with a proper choice of their coherence time - in crystals [32].

For this study we focus on a model two-dimensional system with periodic circular interfaces, which is representative of periodically nanoporous or nanocomposite thin films, a geometry which is most used in micro-technology and in meta-materials. In our recent works cited above, we investigated a silicon-based amorphous matrix with embedded crystalline circular inclusions with different rigidities, using Molecular Dynamics (MD) simulations combined with experimental meaurements. Here, we study the propagation of acoustic wave-packets in the same systems but at a continuum level, to get insight on in-plane thermal transport and acoustic propagation in nanocomposite, as well as nanoporous and nanophononic, thin films. One interest of continuous Finite Element simulations in this case is to allow performing an extensive parametric study on large samples and over sufficiently long simulation times. The price to pay is loosing the acoustic dispersion due to atomic discretization as well as the detail of the scattering sources at the atomic scale. However, these effects were already investigated with MD simulations, and will play an already well identified role only when the wavelength of the excitation becomes comparable to the interatomic distance, that is far smaller than the inclusion size here. For this reason, we ignore them in this study, cnocebtrating on the effect of the interfaces on wave-packet propagation.

The paper is organized as follows: in the second part, we describe how the finite element calculations are performed; in the third part, we detail the different dynamical regimes for wave-packet propagation in our 2D composite model material; in the fourth part, we focus our attention on the determination of mean-free path and attenuation lengths; finally, we discuss the results and we conclude in the last part.

## 2. Numerical Tools

**Figure 2:**
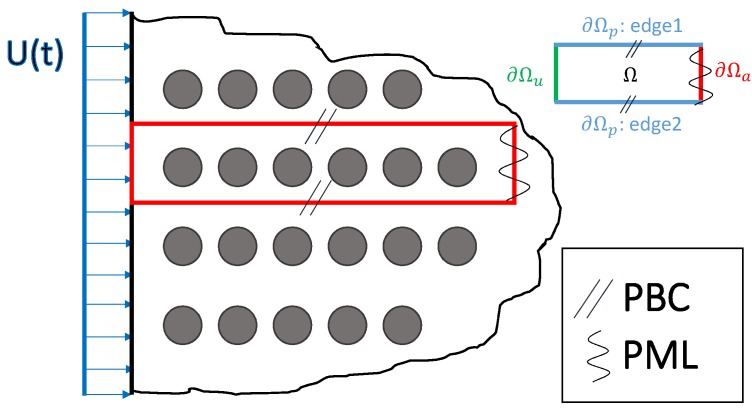
2D simulation model of a solid with circular inclusions: this semi-infinite solid can be represented by only modeling only the part inside the red rectangule with Periodic Boundary Conditions (PBC) and Perfect Matched Layers (PML) as drawn; Grey disks represent the inclusions. (Ω represents simulation domain, ∂Ω indicates boundary conditions)

**Figure 3:**
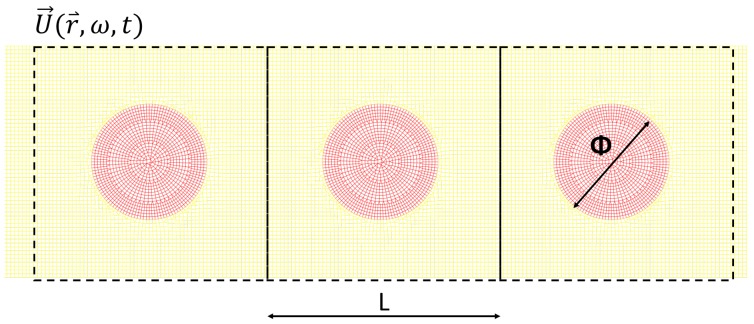
Schematic illustration of the central part of the sample for L = 60 Å: in yellow represents the matrix material, in red the inclusions. Periodic reproduction of this sample along the vertical direction is assured by periodic boundary conditions.

We used Finite Element numerical calculations to study the vibrational properties of a 2D semi-infinite elastic system with circular inclusions positioned along a cubic lattice (Figure 2). For wavefronts parallel to the left boundary, this semi-infinite domain can be numerically simplified by looking at a single line of inclusions, and imposing periodic boundary conditions along the vertical direction (see Supplementary Materials for more details). We thus focused our study on a single alignment of inclusions, with periodic boundary conditions (PBCs) imposed at the top and bottom of the sample shown in Figure 3, and with absorbing boundary conditions, or PML (Perfect Matched Layers) on the right side, to avoid waves reflection [33,34,35,36,37]. The technical details about the boundary conditions and discretization schemes used in the calculations, are presented in Appendix A.

The computational model consists of 28 squares, aligned in the horizontal direction: the first two with only the matrix phase, then 25 containing each a single circular inclusion, and finally another one without inclusion. The large number of squares is necessary for establishing the wave-packet and studying the transient behavior during its propagation from left to right. The size of each square is 60 Å× 60 Å, thus determining the distance between inclusions, as inspired by Figure 1, and corresponding to a nanostructuration, as needed for effectively affecting thermal transport. Meanwhile, the radius of the inclusions will be considered as a control variable in this study. The wave-packet is generated imposing a displacement on the left side of the first square, and it is established in the first 2 squares before touching the first inclusions. The following 25 squares with circular inclusions constitute the useful area for the analysis, while the last empty square allows for the implementation of the absorbing boundary conditions.

**Figure 4:**
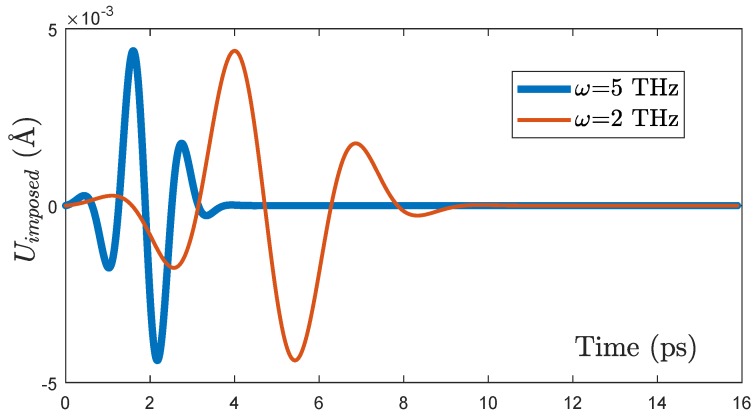
Wave packet imposed as the displacement of the left side of the sample, for two different frequencies: 2 THz, 5 THz

For producing the quasi-monochromatic propagating wave-packet (Figure 4) at the origin of the excitation energy, we impose the displacement on the left side of the sample around x=0 in a small time interval around t=0:

(1) $$U\left(\omega ,t\right)=U_{0}\exp\left(-\frac{{\left(t-3t_{0}\right)}^{2}}{2t_{0}^{2}}\right)\sin\left(\omega t\right)$$

where $U_{0}$ is a constant value, ω is the frequency of this quasi-monochromatic excitation, and $t_{0}$ is the coherence time of the wave-packet. For numerical purposes, it was chosen as $t_{0}=\frac{\pi }{\omega }$, that is half period of the excitation. A displacement parallel to the boundary corresponds to a transverse excitation, while the one perpendicular to the boundary to a longitudinal excitation. For the sake of simplicity, we will consider here only longitudinal excitations. The amplitude $U_{0}$ has been chosen as $U_{0}=4.9\times 10^{-3}$ Å similarly to what was done in our previous MD simulations of propagating wave-packets [38], to prevent anharmonic effects. This choice will also allow for further comparisons of the results with the cited work. A special mention deserves the choice of the coherence time. $t_{0}=\frac{\pi }{\omega }$ is rather a short time, which has been chosen for avoiding the mixing of different plane waves in the amorphous matrix, as explained in the introduction. This however results in a quite broad phonon bandwidth when the Fourier Transform is performed, leading to a monochromaticity of the order of Δω/ω=0.6, questioning the pertinence of this wave-packet for representing acoustic phonons and their contribution to thermal transport. To rule out any possible doubt about this point, we have also investigated larger coherence times, as reported in the Supplementary Materials, giving a monochromaticity up to Δω/ω∼0.2. Our study reveals that the same results are recovered not only on the penetration length but also on the identification of different energy transport regimes, confirming thus the pertinence of the chosen short-time wave-packet for getting insight into energy transfer in this material.

Like in our previous (MD) works [38,39,40], the matrix material is a model amorphous silicon, linearly elastic with isotropic homogeneous elastic behavior characterized by the Young’s modulus $E_{m}=92.25$ GPa, the mass density ρ=2303 kg/m${}^{3}$ and the Poisson ratio ν=0.34. For the inclusions, the Poisson’s ratio is supposed to be the same, while the Young’s modulus $E_{i}$ is taken as another control variable and defined as $E_{i}=E_{m}\times \frac{E_{i}}{E_{m}}$, this latter being the stiffness ratio that was tuned from 0.2 to 10. Table 1 summarizes the values of the parameters used in this work:

Among all the FEM time integration schemes, Newmark scheme [41] is the most common solution for a dynamic problem. We have selected the central-difference algorithm from the Newmark scheme family with parameters $\gamma =\frac{1}{2}$ and β=0. These chosen values of γ and β make sure that once a good time step is determined for a set of meshing values, our calculation will always be converged with a discrete symplectic framework. We can thus perform an extensive parametric study without re-estimating the time step, and with a very good level of accuracy, robustness and efficiency. To satisfy the physically based criteria reported in the Appendix, Equations (A8) and (A9), the chosen step time is 1.58926 fs and the simulations ends at 15.58926 ps.

## 3. Vibrational Properties

### 3.1. Equilibrium Vibrational Properties

**Figure 5:**
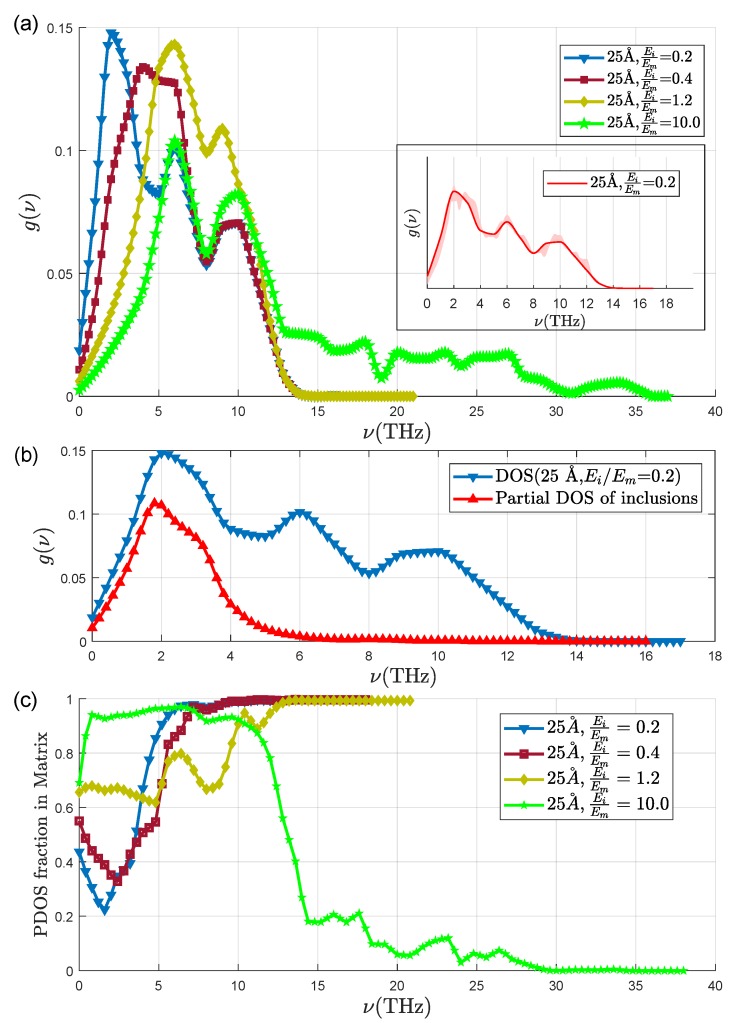
From top to bottom: (**a**) The VDOS for r=25 Å and $\frac{E_{i}}{E_{m}}=0.2,0.4,1.2,10.0$ at discrete frequencies ν with width Δν = 1 THz. Inset: VDOS vs ν for $\frac{E_{i}}{E_{m}}=0.2$ and r=25 Å. The red line corresponds to Δν = 1 THz, while the shaded region to a Δν varying between 0.4 and 1 THz (**b**) VDOS (blue) and partial DOS of inclusions (red) for r=25 Å and $\frac{E_{i}}{E_{m}}=0.2$ (**c**) Fraction of VDOS supported by the matrix for r=25 Å and different values of $\frac{E_{i}}{E_{m}}$, at discrete frequencies ν with Δν=1 THz (Triangle, square, diamond and pentagram symbol are used to indicate $\frac{E_{i}}{E_{m}}=0.2,0.4,1.2$ and 10.0 respectively).

**Figure 6:**
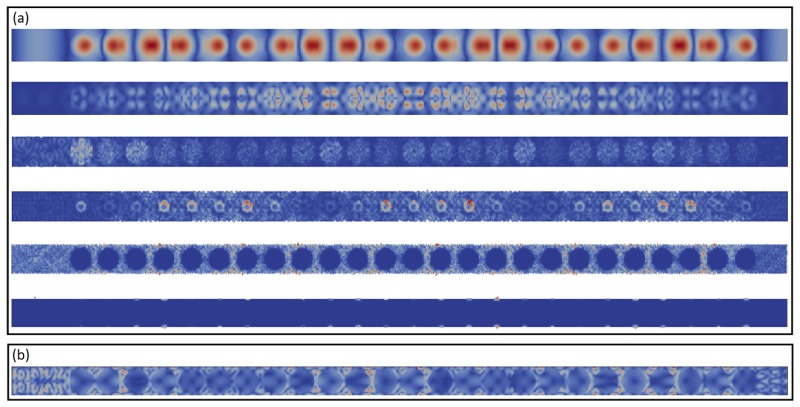
(**a**) Visualization of the normal modes obtained in the system with $\frac{E_{i}}{E_{m}}$ = 0.2 and r=25 Å. The frequencies of the characteristic normal modes (from top to bottom) are: ν = 0.325195 THz (ω = 2πν = 2.04 THz); ν = 0.790191 THz (ω = 4.96 THz); ν = 2.00000 THz (ω = 12.57 THz); ν = 3.99998 THz (ω = 25.13 THz); ν = 6.00019 THz (ω = 37.70 THz); ν = 14.22239 THz (ω = 89.36 THz). (**b**) Visualization of one normal mode obtained in the system with $\frac{E_{i}}{E_{m}}$ = 10 and r=25 Å. The frequency of the mode is: ν = 2.00281 (ω = 12.58 THz).

We first characterize the vibrational response of our systems at equilibrium, before applying a traveling wave packet. We can compute the vibrational density of states (VDOS) of the system and its vibrational modes by performing a spectral analysis, consisting in diagonalizing the dynamical matrix. The eigenvectors of the dynamical matrix are the vibrational eigenmodes (also called normal modes), and the eigenvalues are the squared frequencies of the related vibrations [12]. This is done automatically using the command *VIBR* in Cast3M on successive finite ranges of frequencies. The VDOS is then obtained by counting the number of eigenfrequencies in the intervals with width Δν around each frequency ν. In Figure 5a we report the DOS for several rigidity contrasts ranging from 0.2 to 10, calculated with Δν=1 THz. In order to estimate the resolution of our calculation on the basis of the chosen frequency interval width, we have calculated the DOS for different Δν, going from 0.4 to 1 THz, as reported in the inset. It can be seen that the Δν=1 THz choice is reasonable and does not deteriorate too much the resolution. As usual in 2D systems dominated by plane waves, the VDOS starts with a ∝ω behavior, but, due to the presence of inclusions in our systems, it is then a non-monotonous function of the frequency. It is interesting to spatially solve the VDOS and separate the contribution of the matrix from the one of the inclusions. This is shown in Figure 5b, for $\frac{E_{i}}{E_{m}}=0.2$ and r=25 Å. Being the inclusion much softer, its vibrational modes are softer than the ones of the matrix, and indeed its contribution to the VDOS is limited to frequencies smaller than ≈6 THz. The relative ratio of the matrix to the inclusions contribution to the VDOS is reported in Figure 5c for r=25 Å and different values of $\frac{E_{i}}{E_{m}}$. It is clear that the frequencies corresponding to a specific response of the inclusions progressively increase with the inclusion rigidity. In the most rigid case, when $\frac{E_{i}}{E_{m}}=10$, the high frequency modes are mainly supported by the inclusions, while in the other cases, the response of the inclusions is mainly located below 6 THz. Only for a very weak rigidity contrast, such as $\frac{E_{i}}{E_{m}}=1.2$, it is not possible to clearly identify a specific range for a dominating contribution from the inclusions. From this, we can conclude that by exploring rigidity contrasts ranging from 0.2 to 10, we are able to observe a phenomenology corresponding to all possible situations: from the case where the low frequency modes are mainly supported by the inclusions (low $\frac{E_{i}}{E_{m}}$) up to the case where the vibrations of the inclusions are at frequencies higher that the response of the matrix ($\frac{E_{i}}{E_{m}}=10$). Figure 6 allows to visualize the spatial distribution of the eigenmodes for different frequencies and rigidity contrasts. The low frequency vibrations for $\frac{E_{i}}{E_{m}}=0.2$ are clearly supported by the inclusions, that appear to become mute at high frequency, while the matrix dominates the response at low frequency in the most rigid system with $\frac{E_{i}}{E_{m}}=10$. If the effect is stronger for stronger rigidity contrasts (0.2 and 10), in all cases the presence of the inclusions perturbs the eigenmodes, giving rise to a very inhomogeneous spatial distribution, in some frequency ranges. The question thus arises how such complex spatial vibrational heterogeneity can affect the transient propagation of traveling wave-packets in our systems.

### 3.2. Wave-Packet Propagation: Different Regimes

As said before, the wave-packet is created by imposing a displacement on the left side of the sample. Its propagation is then followed along the sample, in the *x* direction. Due to the presence of interfaces, and related spatial inhomogeneities, the wave-packet wave-vector $\vec{k}$ does not remain constant, the wave-packet being scattered by the inclusions. To understand how such scattering affects the energy transfer, we measure the envelope of the kinetic energy induced in the system by the propagation of the wave-packet, averaged over the y-direction. The energy envelope is defined for each excitation frequency ω as

(2) $$P_{\omega }\left(x\right)=\max_{t}E_{k}\left(x,t\right)$$

where $E_{k}\left(x,t\right)$ is the instantaneous kinetic energy supported by the frame located in *x* with width Δx=2 Å.

**Figure 7:**
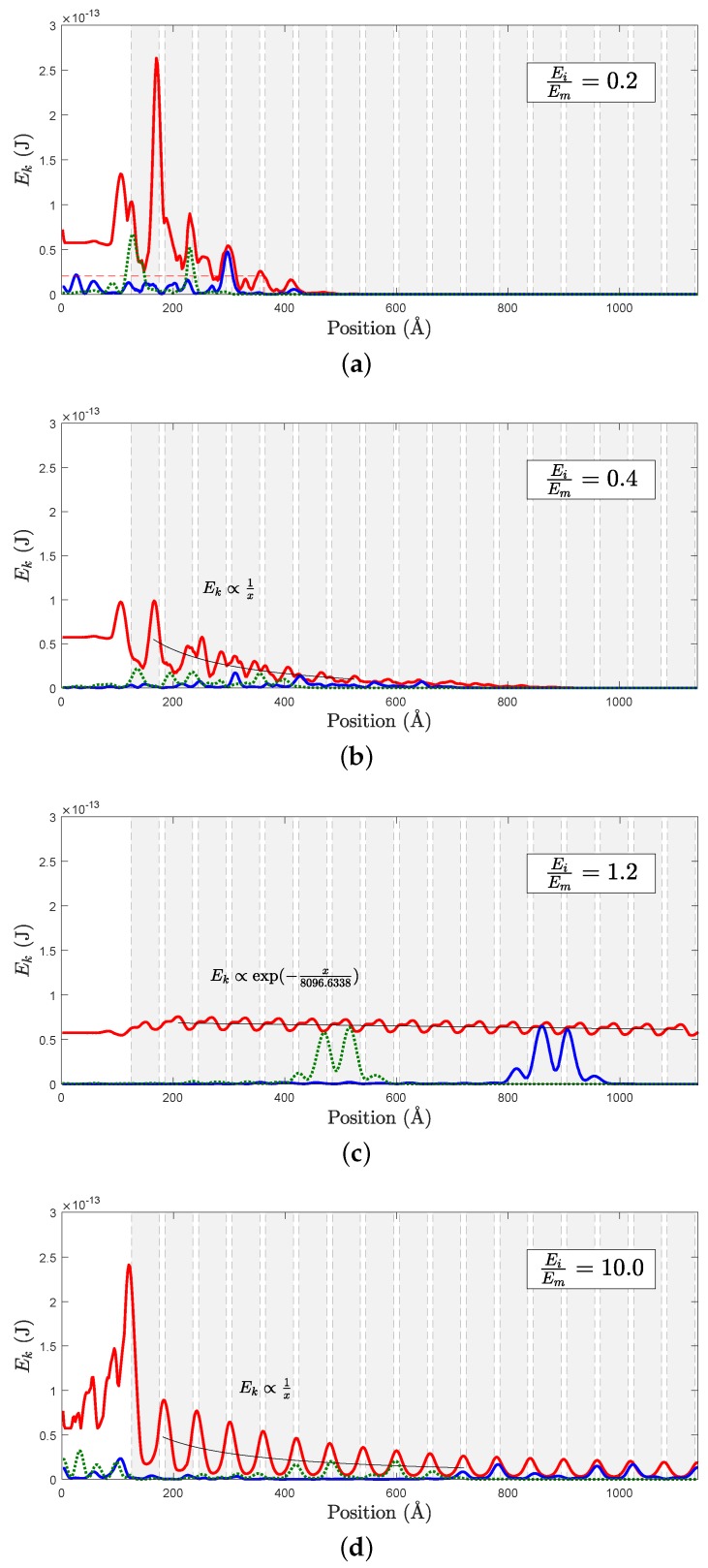
The envelope of kinetic energy for different rigidity contrasts for ω=5 THz, r=25 Å is reported as a red line, while the instantaneous distribution of kinetic energy is given at t = 7.9463 ps (green dotted line) and at t = 12.7174 ps (blue line). Gray areas delimited by dotted lines indicate the positions of the circular inclusions.

As an example, we report in Figure 7 the kinetic energy envelope (red curve) for r=25 Å at a wave-packet frequency of ω=5 THz, for different rigidity contrasts. Instantaneous wave-packets (green and blue curves) are also shown for clarity. The positions of the inclusions are indicated by the gray area delimited by dotted lines. It is clear that the envelope presents oscillations correlated with the presence of interfaces. Moreover, the inclusions cause a more or less efficient attenuation of the kinetic energy along the main direction of propagation. Since the simulations are performed at constant energy (no damping term in the numerical scheme), the observed attenuation is not related to a global dissipation of energy, but it is due to a redistribution of the kinetic energy in directions different from the one of propagation, so that, when averaging this effect along the transverse y-direction, an effective attenuation along the x-direction appears. The strength of this attenuation depends on the different control parameters, such as inclusions size, wave-packet frequency and rigidity contrast. For example, in case of a weak rigidity contrast (Figure 7c) a global exponential attenuation similar to a Beer-Lambert law is observed [38,42]

(3) $$P_{\omega }\left(x\right)\propto \exp\left(-x/\Lambda (\omega )\right)$$

while for larger rigidity contrasts (Figure 7b,d), the algebraic attenuation of the envelope

(4) $$P_{\omega }\left(x\right)\propto 1/x$$

is the signature of a diffusive process [38]. Finally, in the case of very soft inclusions ($E_{i}=0.2E_{m}$, Figure 7a), the attenuation is extremely efficient and the energy seems pinned at the interface between the first inclusion and the matrix, on the inclusion’s side.

**Figure 8:**
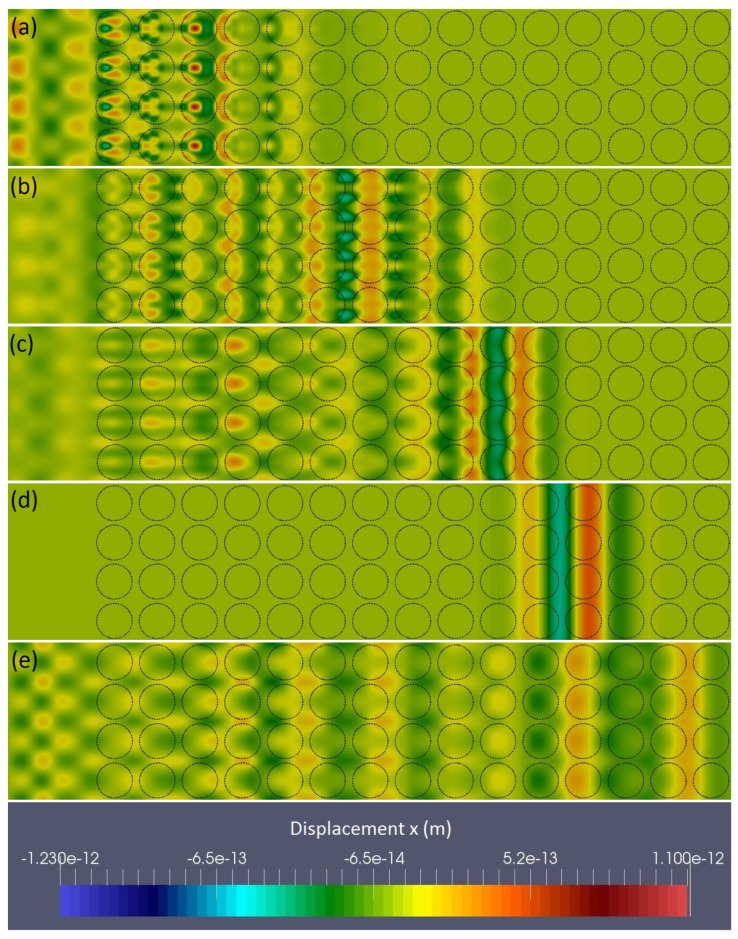
Snapshots of 5 cases at the same time ($t=1.27171\times 10^{-11}$ s) with ω=5 THz and r=25 Å (dashed cirles indicate the positions of the inclusions) (**a**) $E_{i}/E_{m}=0.2$ (**b**) $E_{i}/E_{m}=0.4$ (**c**) $E_{i}/E_{m}=0.6$ (**d**) $E_{i}/E_{m}=1.0$ (**e**) $E_{i}/E_{m}=2.0$.

To confirm our interpretation of the energy envelope behavior, we report in Figure 8 the displacement field for ω=5 THz and different rigidity contrasts. Here we can see that at this frequency for a soft inclusion (Figure 8a) the wave-packet looks pinned inside the inclusions, while for no rigidity contrast ($E_{i}=E_{m}$) it is insensitive to their presence and propagates without any perturbation (Figure 8d). It is worth underlying that all snapshots have been taken at the same time, which clearly shows that the effective velocity of the wave-packet increases with the inclusion rigidity, so that when inclusions are more rigid than the matrix the wave-packet is accelerated (Figure 8e). Moreover, except for the case of no contrast, the wave-packet is spread, and thus attenuated, along the x-direction.

To quantify the energy transfer along the x-direction in all these cases, we calculate the average position of the wave-packet by the weighted arithmetic mean

(5) $$\begin{array}{c} \langle x\rangle \left(t\right)=\frac{\sum_{i}x_{i}E_{k}\left(i,t\right)}{\sum_{i}E_{k}\left(i,t\right)} \end{array}$$

and its spreading around its average position by the standard deviation:

(6) $$\begin{array}{c} \begin{array}{cc} \sigma (x,t) & =\sqrt{\langle {\left(x-\langle x\rangle \right)}^{2}\rangle }\\ & =\sqrt{\frac{\sum_{i}\left(E_{k}\left(i,t\right)\times x_{i}^{2}\right)}{\sum_{i}E_{k}\left(i,t\right)}-{\langle x\rangle }^{2}} \end{array} \end{array}$$

where $x_{i}$ is the position of the $i^{th}$ frame with width Δx in the x-direction, and $E_{k}\left(i,t\right)$ is the instantaneous total kinetic energy supported by that frame. The spreading σ(x,t) can also be rewritten

(7) $$\begin{array}{c} \sigma \left(x,t\right)=\sqrt[{}]{R^{2}\left(t\right)-{\langle x\rangle }^{2}} \end{array}$$

by introducing $R^{2}$, the squared width [40] defined as

(8) $$R^{2}\left(t\right)=\frac{\int_{-\infty }^{\infty }x^{2}E\left(\omega ,x,t\right)dx}{\int_{-\infty }^{\infty }E\left(\omega ,x,t\right)dx}$$

In case of a diffusive process, $\sigma^{2}\left(t\right)$ is proportional to the time *t*, with a slope related to the one-dimensional diffusivity.

(9) $$\sigma^{2}\left(t\right)=2D\left(\omega \right)t.$$

**Figure 9:**
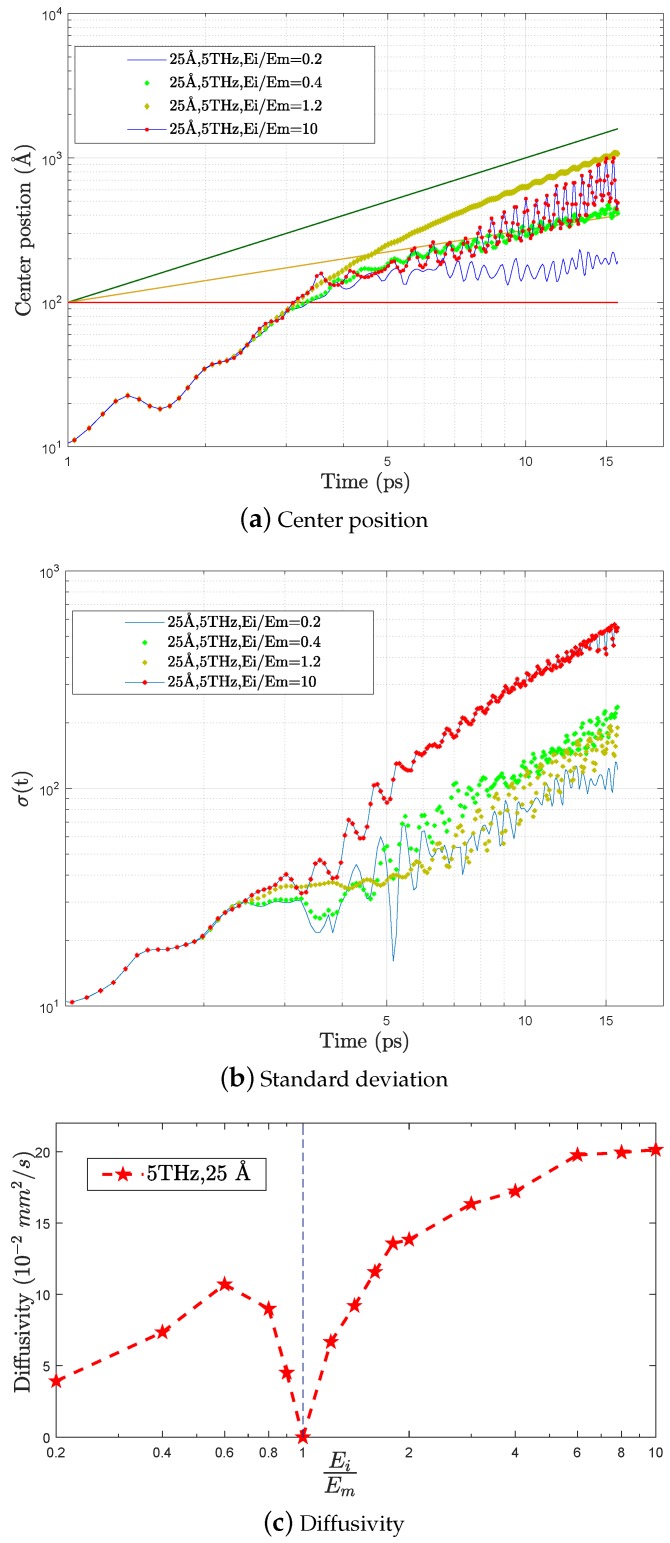
(**a**) Time dependence of the wave packet center position for different rigidity contrasts. We report as well the expected behaviors for propagative regime 〈x〉(t)∝Vt (green line), diffusive regime $\langle x\rangle \left(t\right)\propto {\left(2Dt\right)}^{\frac{1}{2}}$(yellow line) and localized regime 〈x〉(t)∝cst (red line). (**b**) Standard deviation versus time calculated by Equation (6). (**c**) Diffusivity obtained from Equation (9) using an excitation with random polarizations, for different values of the rigidity contrast $E_{i}/E_{m}$.

**Figure 10:**
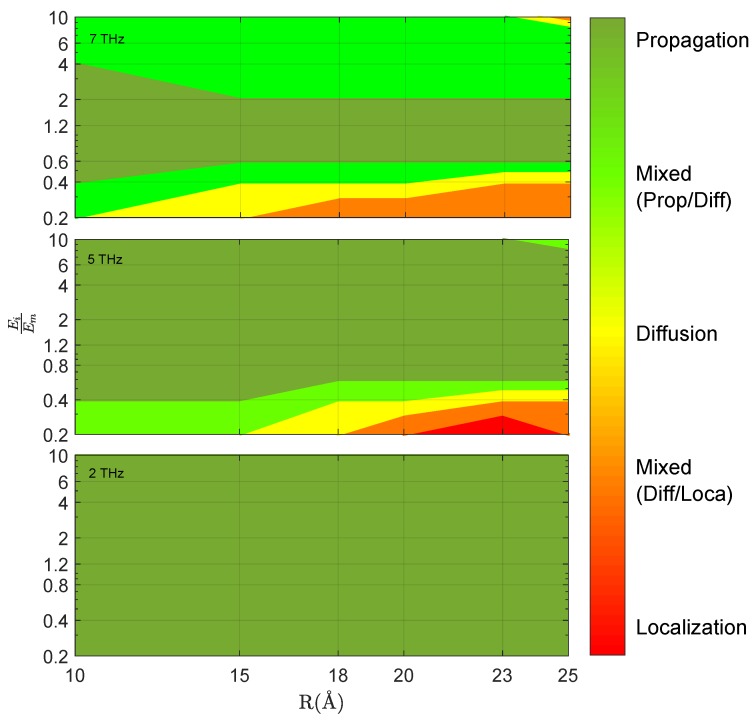
Visualization of the different dynamical regimes of the Wave Packets (propagative, diffusive, localized), as a function of the radius *r* of the inclusions and of the relative rigidity $E_{i}/E_{m}$ for frequencies ω ranging from 2 to 7 THz.

Figure 9a shows a representative set of different regimes of energy transfer: the case $\frac{E_{i}}{E_{m}}$ = 0.4 (green dots) is a clear example of *diffusive* regime where $\langle x\rangle \propto \sqrt{t}$, while $\frac{E_{i}}{E_{m}}$ = 1.2 (yellow dots) yields to a propagative regime ($\langle x\rangle =v_{L}.t$). The other cases shown in this figure are more complex: the most rigid case $\frac{E_{i}}{E_{m}}$ = 10 (red dots with blue line) is initially similar to a diffusive regime, but becomes progressively propagative, while in the very soft case $\frac{E_{i}}{E_{m}}$ = 0.2 (blue line) the wave-packet is initially pinned (〈x〉=cste) and progressively gets unpinned following a diffusive motion. Even if this last regime is not strictly localized, the pinning induces an interesting delay in the energy transfer, that could be of some technological interest when dealing with combined heat processes in nanoscale electronical devices. Note that the largest spreading of the wave-packet takes place in the highest rigidity contrast case, $E_{i}/E_{m}=10$ (Figure 9b), due to the wave-packet acceleration within the inclusions. The different regimes of energy transfer and their dependence on the parameters are summarized in Figure 10: here it can be seen that increasing rigidity contrast and frequency, an increasingly complex behaviour arises. Softer inclusions induce the most complex behaviour over large frequency ranges.

The diffusive spreading of the wave packet can be quantified by the diffusivity parameter *D* (Equation (9)). The dependence of *D* on the rigidity contrast, for ω=5 THz, is shown in Figure 9c. It is globally increasing with ω but exhibits deep minimum for $E_{i}/E_{m}\approx 1$. The most interesting range for reducing energy transfer thus appears to be the low-$E_{i}/E_{m}$ range, where the low diffusivity is not counterbalanced by an important propagative contribution, as it is the case for $E_{i}/E_{m}\approx 1$, where the dominant contribution to energy transfer is the propagative one.

In order to get a quantitative insight on the energy transfers, comparing all situations, beyond the observed variety of dynamical behaviors in presence of inclusions with rigidity contrast, we can define for all cases the spatial attenuation of the energy envelope. This is reported in the next section, where we extend the study mainly reported here for ω=5 THz and r=25 Å, to a large range of inclusions volume fractions and excitation frequencies and perform an extensive parametric study of this spatial attenuation.

## 4. Mean Free Path vs. Penetration Length

Depending on the dynamical regime, the characteristic length describing energy transfer is not unique, which makes it difficult to compare different regimes. For example, in the propagative regime, for which a Beer-Lambert law for the energy attenuation is found, the characteristic length is the mean free path Λ(ω). In the diffusive regime, the energy envelope follows an algebraic decay without any intrinsic lengthscale, which depends only on the initial energy [38]. The ability for heat transfer is then better quantified by the diffusivity (see previous section), which directly enters in the computation of the thermal conductivity, although absent from the attenuation behavior of the envelope. Finally, in the case of an energy envelope pinned over a small length $l_{pin}$, this latter will be the characteristic length. In order to quantify the ability of a system for energy transfer, independently on the dynamical regime, we look at the long-time penetration length, defined as the traveled length above which the energy per unit length remains always smaller than the maximum excitation energy per unit length divided by *e*. This definition allows us to take into account the presence of oscillations in the energy envelope, which would mine the standard definition of penetration length. Concerning the three regimes cited, this length will correspond to the mean free path in the propagative case, be very close to $l_{pin}$ for a pinned energy envelope, and be representative of the energy spread in the sample in the diffusive case. We have calculated the penetration length $l_{p}$ as a function of the inclusion radius, ranging from 10 to 25 Å (corresponding to volume fractions from 8.7% to 54.5% in 2D), of the rigidity contrast, ranging from 0.2 to 10, and of the excitation frequency, ranging from 2 THz to 14 THz (along the common part of the vibrational spectrum Figure 5).

**Figure 11:**
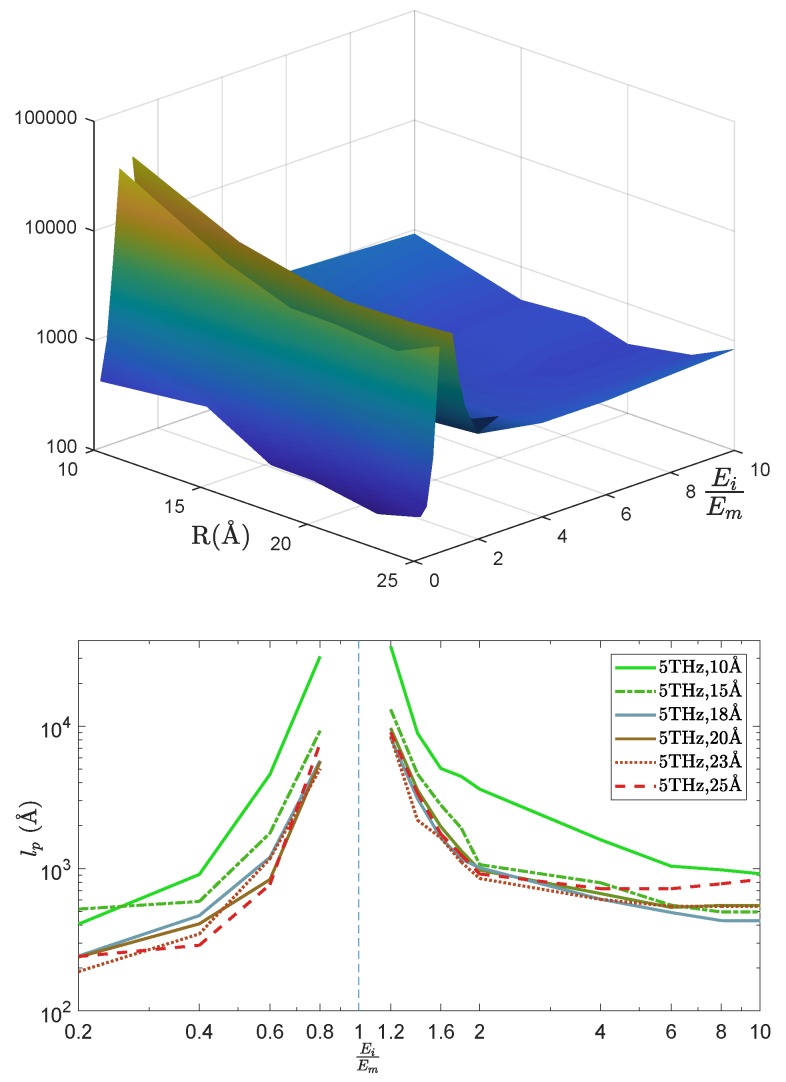
Penetration length $l_{p}\left(\frac{E_{i}}{E_{m}},r\right)$, with $\frac{E_{i}}{E_{m}}$ the stiffness ratio and *r* the radius of inclusion for the frequency of wave packets ω = 5 THz.(Top: 3D Bottom: 2D)

In Figure 11 we report the radius and rigidity contrast dependence for a wave-packet of frequency 5 THz, as representative for all frequencies. First, it can be seen that the penetration length decays with the radius *r* of the inclusion, that is, with the size 2πr of the interface. The penetration length reaches a minimum close to the large radius r=25 Å. For larger radii, the inclusions will be separated by less than a few angstroms, almost touching each other, thus opening a continuous path for the acoustic energy transfer and consequently increasing the attenuation length $l_{p}$.

Second, the penetration length also strongly decreases with the rigidity contrast, but there is a clear asymmetry between the case $E_{i}>E_{m}$ and the case $E_{i}<E_{m}$. Indeed, the more rigid inclusions induce an acceleration of the acoustic waves (larger sound velocity as shown in Figure 8) together with a marked broadening of the wave-packet (Figure 9b), and these two effects result in an increased penetration length. It can be concluded that for the same rigidity contrast (same ratio of acoustic impedances), the softer inclusions will more efficiently limit the penetration of the wave-packet inside the sample. However, the penetration length is not strictly a monotonous function of the rigidity contrast, but goes through a minimum, whose value depends on the excitation frequency. For ω=5 THz and r=25 Å for example, two minima can be found, close to $E_{i}/E_{m}=4$ and $E_{i}/E_{m}=0.4$. Note that in all cases, the attenuation length remains larger than the inclusions size, approaching the distance between three inclusions at its minimum value.

**Figure 12:**
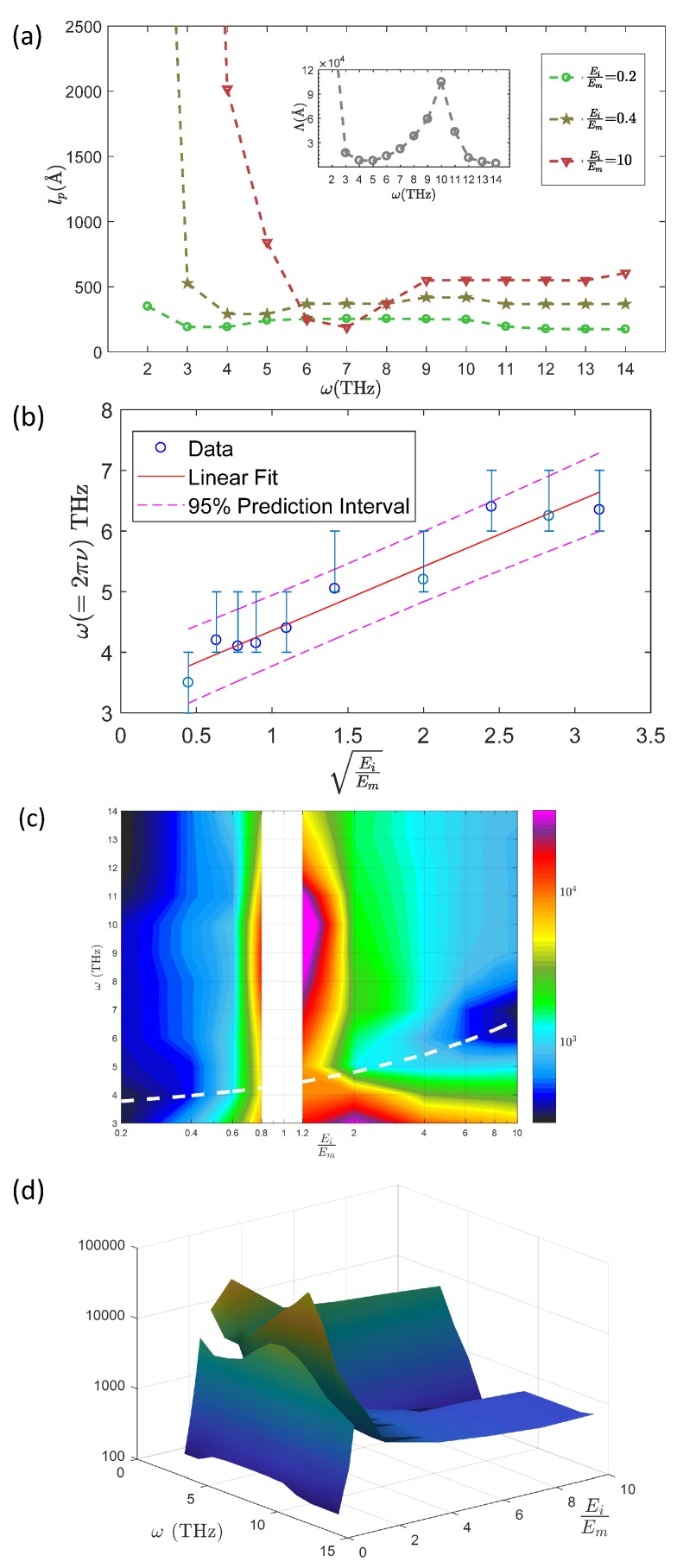
(**a**) Penetration length $l_{p}\left(\omega \right)$ for $\frac{E_{i}}{E_{m}}$ = 0.2, 0.4 and 10.0, with *r* = 25 Å. Inset: mean free paths Λ vs ω for $\frac{E_{i}}{E_{m}}$= 1.2 and r=25 Å. (**b**) Frequency, which corresponds to the minimum $l_{p}$ for each $\frac{E_{i}}{E_{m}}$ as a function of the sound velocity in the inclusions. (**c**) color representation of the penetration length in the parametric space $\left(E_{i}/E_{m},\omega \right)$ (**d**) Idem in a 3D plot. (**d**) 3D plot of the prenetration length in the arametric space $\left(E_{i}/E_{m},\omega \right)$

Despite for the reported case the penetration length for a more rigid inclusion is always longer than for soft inclusions, it is however possible to reverse the situation and get a more efficient sound attenuation with rigid inclusions if another excitation frequency is used. Indeed, as shown in Figure 12a, the penetration length depends on the frequency and, for rigid inclusions, displays a clear minimum at 7 THz. This effect is due to a resonance of the inclusions that will keep the acoustic energy. Indeed, as seen in Figure 12b, the frequency of the minimum in the penetration length evolves linearly with the sound velocity within the inclusion, meaning that inclusions specific vibrations play a key role in determining the frequency dependence of $l_{p}$. This resonant effect is reported in the Figure 12c with white long-dashed line showing the proportionality in the minimum frequency with the sound velocity (in log-linear scale). A 3d plot (Figure 12d) underlines this non-monotonous dependence of the penetration length on the frequency, especially in the high rigidity case that appears to be the most selective with the frequency.

To conclude this part, our numerical measurements have allowed us to definitely assess the following: (i) far from a resonance of the inclusions, the penetration length will decrease with the interface size, provided that there is no inclusions percolation, (ii) the attenuation is globally more efficient for softer inclusions, apart from specific excitation frequencies. The dependence of the penetration length on the three investigated parameters (interface size, rigidity contrast and excitation frequency) is however complex and not monotonous, although it exhibits a sufficiently smooth behavior. It should thus exist a continuous trajectory in the three-parameter space relating local minima of the penetration length, and suggesting an efficient optimization of the acoustic attenuation.

## 5. Discussion

In the previous parts, we have shown that wavepackets dynamics results from a complex mixture of scattering, resonances and propagation, and we have build a frequency dependent phase diagram for different dynamical regimes based on the effective time dependence of energy transportation: propagative, diffusive, localized or mixed (Figure 10). The departure from the propagative regime is globally more pronounced for large rigidity contrasts (especially for softer inclusions) and for high frequencies (when the incident wavelength becomes comparable to the inclusions size). This effective behaviour results from various phenomena: scattering at matrix/inclusion interfaces, waves interference, waves reflexion on curved surfaces inducing billiard motion, gallery modes along the interfaces, or acoustic resonances of the inclusions. In any case, the resulting transfer of energy belongs in a first approximation to one of the four dynamical regimes mentioned above. The deviation from the initial direction of propagation yields to an effective attenuation of the energy in the direction of propagation, also described as an apparent dissipation in the transient regime.

Our parametric study on apparent attenuation of kinetic energy transfer could now be used to optimize different properties: for example the acoustic attenuation at a given frequency, but also the diffusivity of kinetic energy, and thus the thermal conductivity. In the propagative regime, the thermal conductivity results from the product of the attenuation length characteristic of scattering processes (Figure 12) with the heat capacity $C_{v}$ and the group velocity $v_{g}$, integrated over all frequencies as in the following formula [12]:

(10) $$\kappa_{T}\left(T\right)=\int \;d\omega g\left(\omega \right)C_{v}\left(\omega ,T\right)v_{g}\left(\omega \right)l_{p}\left(\omega \right)$$

while in the diffusive regime [13], it is given by the diffusivity (Figure 9) as

(11) $$\kappa_{T}\left(T\right)=\int \;d\omega g\left(\omega \right)C_{v}\left(\omega ,T\right)D\left(\omega \right)$$

It is shown here that systems with softer inclusions have more effect on a global attenuation averaged over all frequencies, thus contributing clearly to decrease the thermal conductivity. The same conclusion holds for the diffusivity that increases with the inclusions rigidity when the diffusive regime matters.

The continuum mechanics description of nano-composite materials used here is clearly a simplified description of real systems, both perfectly periodic nanophononic materials and nanocomposites with randomly distributed inclusions such as the one in Figure 1, since only interfacial effects on the acoustic attenuation and thermal diffusivity are here taken into account. Other scattering sources such as electrons, anharmonicity, thermal activation, or disorder are all neglected, as well as the acoustic dispersion and the local anisotropy due to atomic discretization [43] as already mentioned in the introduction. Despite such well-circumscribed simplifications, our parametric study uses continuum calculations to catch the physics behind energy transport in nanophononic materials and can serve as a guide for materials design and optimization. The model can be used as a basis that could be improved to take progressively into account additional effects. Looking at Figure 12 for example, it is clear that our material can be optimized for energy transport enhancement or inhibition, by choosing the parameters in order to enhance or reduce the acoustic attenuation (the same for the diffusivity) at a given wave-packet frequency. Interestingly, the figure suggests also that such a system could be used as acoustical spectrometer: the attenuation length shows a marked decrease at a characteristic frequency depending on the rigidity ratio, especially in the case of tougher inclusions.

The generalisation of our results to amorphous-based nanophononic or nanocomposite materials, such as the one reported in Figure 1, involves a strong additional energy dissipation due to structural disorder, even in the absence of interfaces [20,38]. Such materials being today at the focus of an intense research for technological applications, it will be of interest to extend first the present parametric study to amorphous/nano-crystalline composites, in order to assess the thermal transport regime in presence of a strong intrinsic energy dissipation together with the artificially introduced (through the nanostructuration) dissipation channel. This will likely require a continuum description of the materials mechanical properties using a frequency dependent visco-elastic constitutive law [44,45].

## 6. Conclusions

In this paper, we have seen in detail the effect of mechanical (rigidity contrast) and geometrical (volume fraction) parameters on the properties of vibrational energy transfer in a nanocomposite material. The goal of our study was to perform a systematic and detailed parametric study of this apparent attenuation, using modelisation tools related to continuum mechanics approaches. Our numerous finite element calculations have revealed that the dependence of the attenuation length on the different parameters is far from simple, and exhibits the following non monotonous behaviours: (1) with respect to the volume fraction, and thus the interface area, suggesting the existence of optimal inclusions radius and interface area, sufficiently large to allow for an efficient scattering but sufficiently low to prevent any energy percolation between the inclusions; (2) with respect to the rigidity contrast. Softer inclusions clearly appear as more efficient for energy attenuation, but the rigid inclusions are also able to pin the vibrational energy at specific frequencies, and thus to decrease strongly the attenuation length at these frequencies; (3) with respect to the excitation frequency, as due for example to the previously mentioned inclusions resonances, which contribute to pin the energy at a given frequency.

These results could have direct applications for high frequency nanophononic materials as acoustic filters for example. Such an approach is also useful to identify the general trends affecting the phononic contribution to thermal conductivity. It gives however an overestimation of the thermal conductivity, since more quantitative calculation needs to take into account the atomic scale dispersive contribution to the vibrational density of states, together with atomic scale scattering processes controlling the diffusivities above the Ioffe-Regel frequency. Within this clearly identified limitation, a systematic backup of the results for given sets of parameters could now interestingly be used to perform real time simulations [46,47].

## Figures and Tables

**Table 1:** List of Parameters and Reference dimensions

$E_{m}$	$\nu_{m}$	*L*	ρ
92.25 GPa [39]	0.34 [39]	60 Å	2303 kg/m${}^{3}$ [39]
$E_{i}/E_{m}$	$\nu_{i}$	*R*	ω
0.2 to 10	0.34	10 to 25 Å,	2 to 15 THz

## Supplementary Materials

The following are available online at https://www.mdpi.com/2079-4991/9/10/1471/s1.

## Appendix A. Finite Elements Simulations

In this section, we detail the numerical method including the excitation of wave packet, the boundary conditions and the dynamical scheme used in the numerical implementation.

### Appendix A.1. Wave Packet

We have studied the vibrational properties of a model system with circular inclusions, whose materials properties are taken from [40]. To study the propagation of the vibrational energy, we excite a quasi-monochromatic wave packet on one side of the sample around x=0 in a small time interval around t=0 [40]. To excite the vibrations in the sample we used the excitation displacement at $\partial \Omega_{u}$

(A1) $$U\left(\omega ,t\right)=U_{0}\exp\left(-\frac{{\left(t-3t_{0}\right)}^{2}}{2t_{0}^{2}}\right)\sin\left(\omega t\right)$$

with $U_{0}$ is a constant value, ω is the frequency of this quasi-monochromatic excitation, $t_{0}=\frac{\pi }{\omega }$, and $U_{0}=4.9\times 10^{-3}$ Å in agreement with [38].

### Appendix A.2. Boundary Conditions

We have applied two boundary conditions in our finite element calculation. As shown in Figure 2, one is periodic boundary condition (PBC) at $\partial \Omega_{p}$, and another is absorbing boundary conditions, or Perfect Matched Layers (PML) at $\partial \Omega_{a}$.

The periodic boundary condition reads:

(A2) $$u_{\eta }{{|}_{edge1}=u_{\eta }|}_{edge2},\;\eta =x,y$$

with u is the nodal displacement. The PBC is realized by using the method of Lagrange multipliers.

The PML detailed in [33,34,35,36,37] results from the creation of a viscous boundary that can absorb the incident wave and prevent the reflected wave. With this method, only a finite number of nodal points need to be considered; thus, an infinite system may be approximated by a finite system with a special viscous boundary condition. On this viscous boundary, the normal stress ζ and the shear stress τ are related to the perpendicular velocity and the tangential velocity of the incident wave respectively. It can be written as:

(A3) $$\begin{array}{cc} \zeta & =a\rho V_{p}{\dot{u}}_{x} \end{array}$$

(A4) $$\begin{array}{cc} \tau & =b\rho V_{s}{\dot{u}}_{y} \end{array}$$

with $V_{s}=\sqrt{\frac{G}{\rho }}$ is the S-wave celerity and $V_{p}=\frac{1}{s}V_{s}=\sqrt{\frac{E}{\rho }}$ is the P-wave celerity, $s^{2}=\frac{1-2\mu }{2(1-\mu )}$, μ is Poisson ratio, *E* and *G* are elastic moduli, *u* and *v* represent the displacement caused by the P-wave and S-wave respectively. Lysmer and Kulemeyer [33] have proposed to use a=b=1, which has been verified as having a very nice performance [35] to absorb quasi-perfectly elastic harmonic waves.

For a 1D problem u(x,t), only (A3) is considered. Replace $\zeta =E\frac{\partial u}{\partial x}$ and a=1, the PML (A3) reads:

(A5) $$V_{p}\frac{\partial u}{\partial x}-\frac{\partial u}{\partial t}=0$$

The corresponding weak form for 1D dynamic problem ($\frac{\partial^{2}u}{\partial t^{2}}=V_{p}^{2}\frac{\partial^{2}u}{\partial x^{2}}$) together with the PML reads [36]:

(A6) $$\begin{array}{cc} \int_{0}^{L}\left(E\frac{\partial u}{\partial x}\frac{\partial v}{\partial x}+\rho \frac{\partial^{2}u}{\partial t^{2}}v\right)dr & =\frac{E}{V_{p}}\frac{\partial u}{\partial t}\left(L,t\right)v\left(L\right)\\ & ,\forall x\in [0,L] \end{array}$$

where *v* is a test function. Displacement loading (wave packet) is charged at x=0, and the PML is at x=L. In the LHS of Equation (A6), the first term is the virtual internal work and second term is the virtual kinetic work. Notice that there is an additional term $\frac{E}{V_{p}}\frac{\partial u}{\partial t}\left(L,t\right)v\left(L\right)$ in the RHS, which is brought by the PML and can be thought as a local damping.

As a supplementary part to the weak form, the Dirichlet boundary condition is used as it is in Equation (1):

(A7) u(0,t)=U(ω,t)

### Appendix A.3. Time Integration

Among all the FEM time integration schemes, Newmark scheme [41] is the most common solution for a dynamic structure. We have selected the symplectic central-difference algorithm from the Newmark scheme family, in that case $\gamma =\frac{1}{2}$ and β=0. These chosen values of γ and β make sure that once a good time step is determined for a set of meshing values, our calculation will always be converged and ensure discrete balance equations (linear momentum, angular momentum and energy) [48]. We can thus change the dimension of the inclusion and the ratio of rigidity (the ratio of Young’s modulus between inclusions and amorphous matrix in this study) with an automatic estimation of the initial time step; it is convenient for our parametric study. Hughes, Geradin, and Rixen [49] have done a detailed stability analysis of the Newmark scheme family. It shows that in order to ensure the convergence, the time step *h* and the natural frequency ω must to satisfy the condition:

(A8) ωh<2

in the case of central-difference, and

(A9) $$\omega_{max}=\frac{V_{p}}{\lambda_{min}}$$

where the wave celerity $v_{wave}$ is a parameter depending on the propagating medium and $\lambda_{min}$ is the minimum wavelength.

### Appendix A.4. Algorithm

Similar to the 1D problem shown in Equation (A6), we can get the space-time discretized form for a 2D plain strain problem (combined with the central-difference time integrator):

(A10) $$M{\ddot{U}}_{n+1}=F_{n+1}-KU_{n}^{p}-\tilde{C}{\dot{\tilde{U}}}_{n+\frac{1}{2}}$$

Here, *K* is stiffness matrix, *M* is lumped mass matrix, $\tilde{C}$ and $\dot{\tilde{U}}$ are the damping matrix and the velocity vector of the PML, respectively. *F* is a possible nonzero external force vector. The indices *n* and n+1 are successive time steps, and the superscript *p* means predictor:

(A11) $$\begin{array}{cc} U_{n}^{p} & =U_{n}+\Delta t{\dot{U}}_{n}+\frac{\Delta t^{2}}{2}{\ddot{U}}_{n} \end{array}$$

(A12) $$\begin{array}{cc} {\dot{U}}_{n}^{p} & ={\dot{U}}_{n}+\frac{\Delta t}{2}{\ddot{U}}_{n} \end{array}$$

substitute (A11) and (A12) into (A10) and hence find the updated acceleration:

(A13) $${\ddot{U}}_{n+1}=M^{-1}\left(F_{n+1}-KU_{n+1}-\tilde{C}{\dot{\tilde{U}}}_{n+\frac{1}{2}}\right)$$

where:

(A14) $$\begin{array}{cc} U_{n+1} & =U_{n}^{p} \end{array}$$

(A15) $$\begin{array}{cc} {\dot{U}}_{n+\frac{1}{2}} & ={\dot{U}}_{n}^{p} \end{array}$$

(A16) $$\begin{array}{cc} {\dot{\tilde{U}}}_{n+\frac{1}{2}} & ={\dot{U}}_{n+\frac{1}{2}}{|}_{\partial \Omega_{a}} \end{array}$$

Then, the updated velocity is:

(A17) $${\dot{U}}_{n+1}={\dot{U}}_{n+\frac{1}{2}}+\frac{\Delta t}{2}{\ddot{U}}_{n+1}$$

Finally, let n=n+1 and repeat the process from (A11) to (A16) until to the end.
