# Supplementary material for: Thermal Transport in a 2D Nanophononic Solid: Role of bi-Phasic Materials Properties on Acoustic Attenuation and Thermal Diffusivity
Source: Nanomaterials (Basel). 2019 Oct 16;9(10):1471. doi: 10.3390/nano9101471 (PMC6836169; doi:10.3390/nano9101471)
Supplement: Supplementary file 1 [file nanomaterials-09-01471-s001.zip › SupplementaryMaterial/ReadMe-LUO-TANGUY-2019.pdf]

Here is the list of files given in the Supplementary Material fo the article entitled

« Thermal transport in a 2D Nanophononic solid: role of bi-phasic materials properties on acoustic attenuation and thermal diffusivity »
by Haoming Luo, Anthony Gravouil, Valentina Giordano and Anne Tanguy

**Folder « Multi-couche »** contains movies allowing a comparison between wave propagation in samples made with a single line of inclusions, and samples made with a set of parallel lines of inclusions. In the name of the files, ri = Ei/E mis the rigidity ratio, the number in THz is the frequency, and R is the radius of the inclusions in Angströms.

The figure **PenetrationLength.png** allows comparing the penetration length in both cases (single line of set of lines)

The figures **WP_EisEm*.png** show the enveloppe of the wave packets (same notations)

The file **WavePacketsPropagationExamples.pptx** includes movies showing the propagation of a wave packet along a line of insclusions for different values of ri, together with an example of drawing of the enveloppe of a wave packet along its propagation.

Finally, the files **FFT_WP_t0.pdf** and **pene_depth_t0_5THz_r=25.pdf** show the sensitivity of the results at w=5 THz to the coherence time t0 of the wave packet.
