# Supplementary material for: Thermal Transport in a 2D Nanophononic Solid: Role of bi-Phasic Materials Properties on Acoustic Attenuation and Thermal Diffusivity
Source: Nanomaterials (Basel). 2019 Oct 16;9(10):1471. doi: 10.3390/nano9101471 (PMC6836169; doi:10.3390/nano9101471)
Supplement: Supplementary file 1 [file nanomaterials-09-01471-s001.zip › SupplementaryMaterial/WavePacketsPropagationExamples.pptx]

## Slide 1
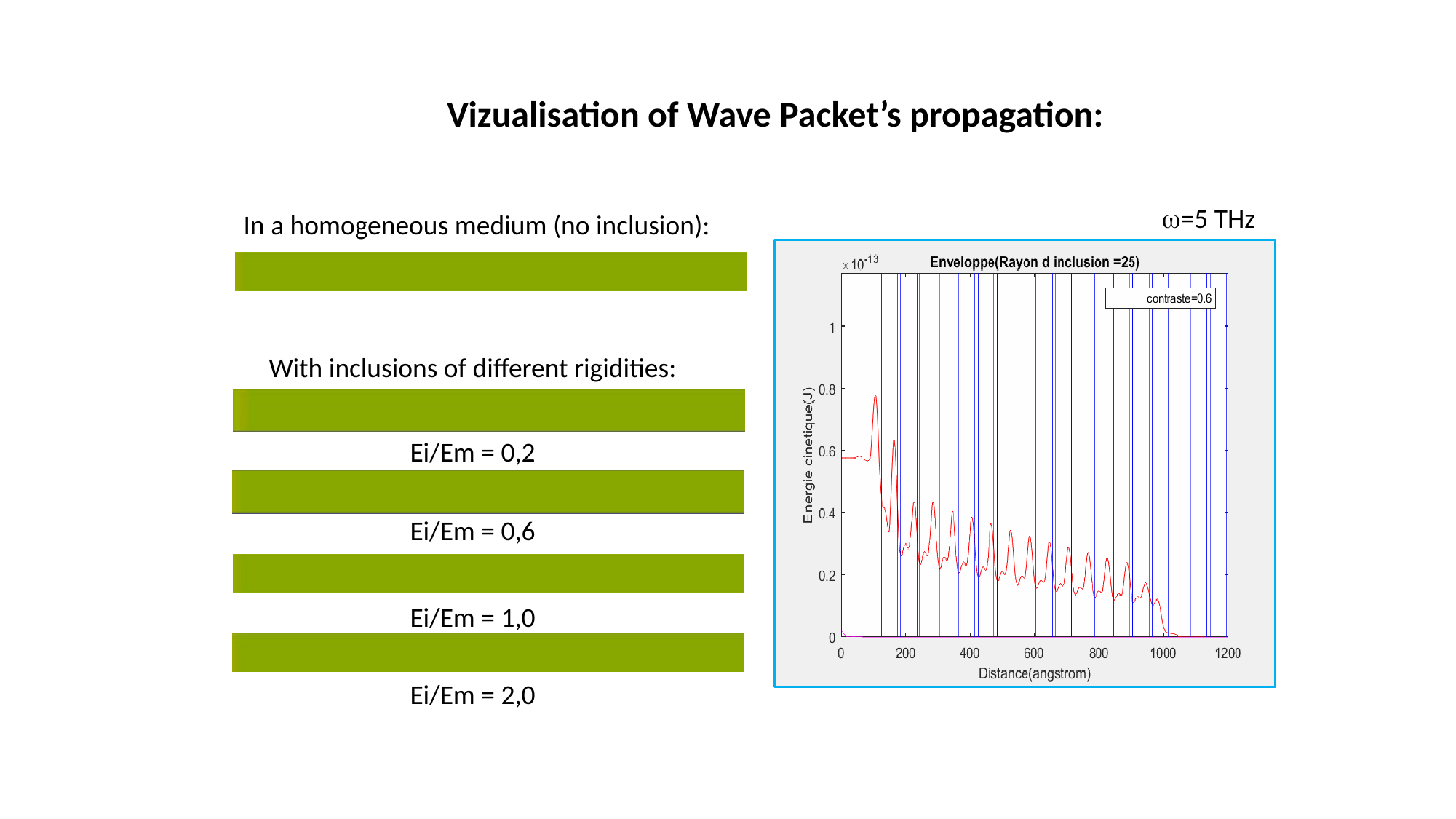

Vizualisation of Wave Packet’s propagation:
w=5 THz
In a homogeneous medium (no inclusion):
With inclusions of different rigidities:
Ei/Em = 0,2
Ei/Em = 0,6
Ei/Em = 1,0
Ei/Em = 2,0
