# Supplementary figures and images for: Thermal Transport in a 2D Nanophononic Solid: Role of bi-Phasic Materials Properties on Acoustic Attenuation and Thermal Diffusivity
Source: Nanomaterials (Basel). 2019 Oct 16;9(10):1471. doi: 10.3390/nano9101471 (PMC6836169; doi:10.3390/nano9101471)

Single-Sided Amplitude Spectrum

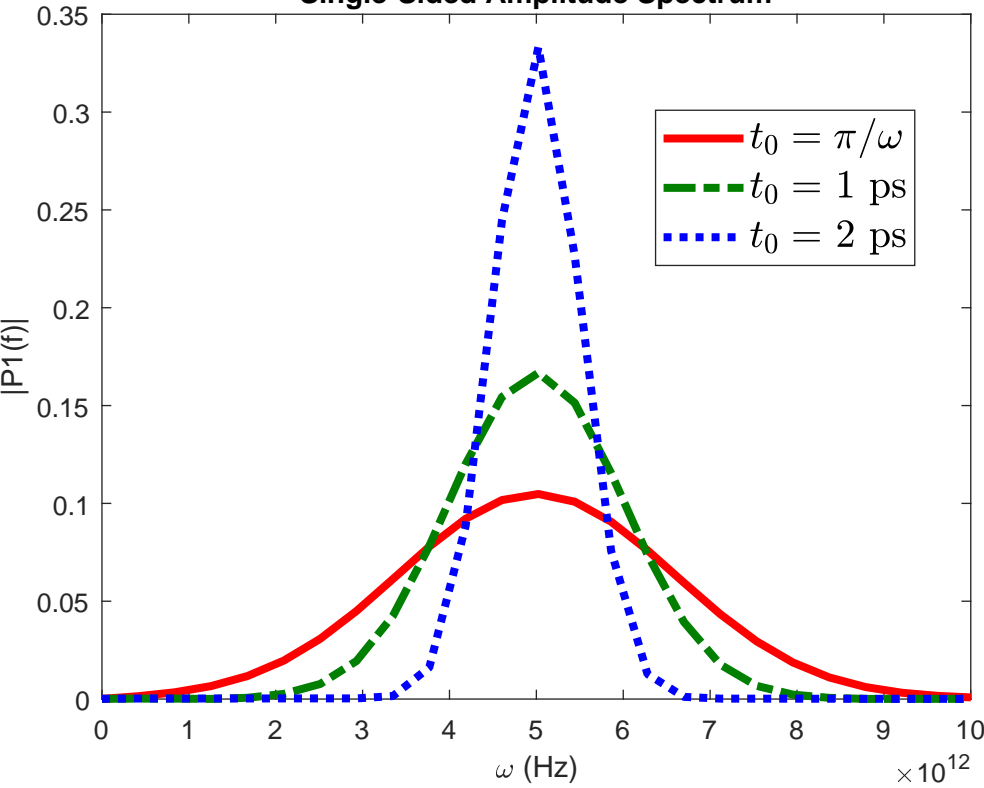

Supplement: Supplementary file 1 [file nanomaterials-09-01471-s001.zip › SupplementaryMaterial/FFT_WP_t0.pdf]

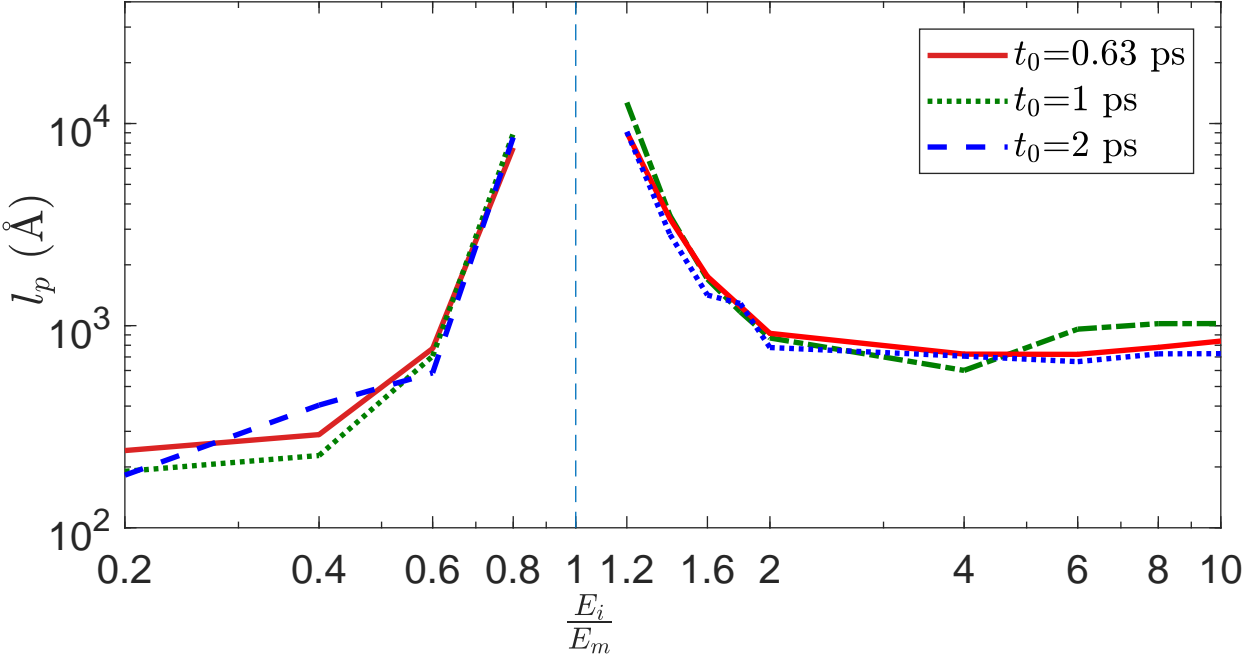

Supplement: Supplementary file 1 [file nanomaterials-09-01471-s001.zip › SupplementaryMaterial/pene_depth_t0_5THz_r=25.pdf]

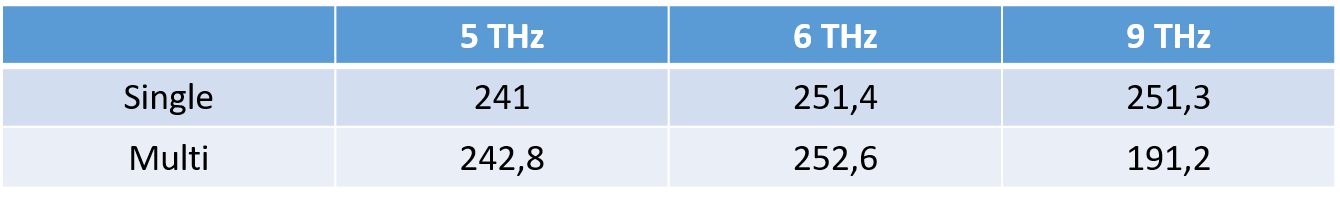

Supplement: Supplementary file 1 [file nanomaterials-09-01471-s001.zip › SupplementaryMaterial/PenetrationLength.png]

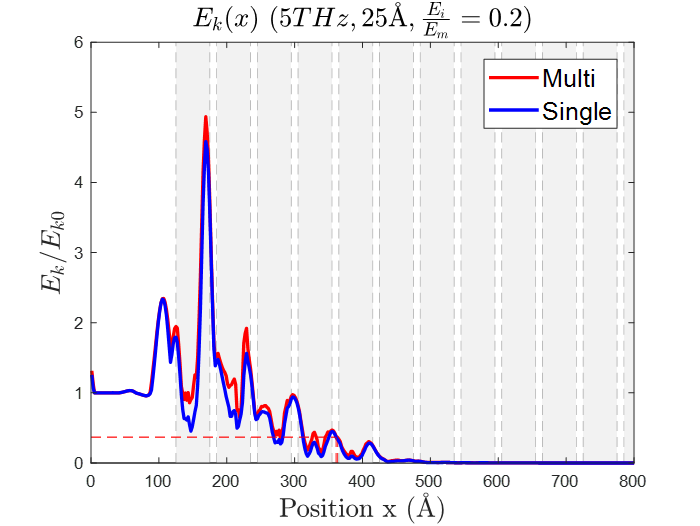

Supplement: Supplementary file 1 [file nanomaterials-09-01471-s001.zip › SupplementaryMaterial/WP_ri=0.2_R=25_5THz.png]

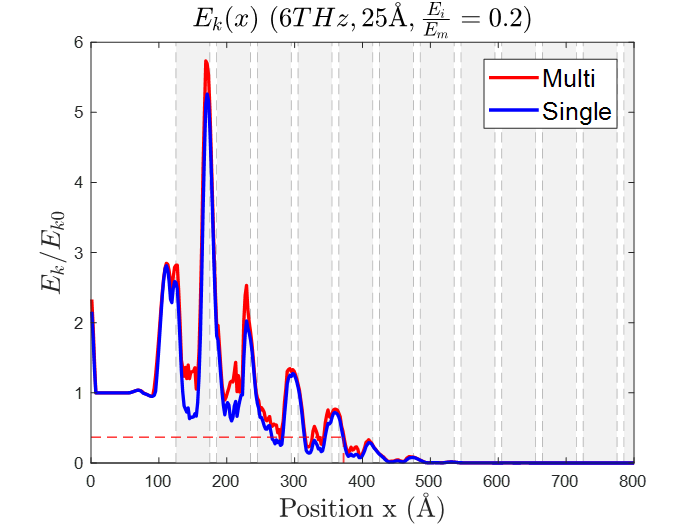

Supplement: Supplementary file 1 [file nanomaterials-09-01471-s001.zip › SupplementaryMaterial/WP_ri=0.2_R=25_6THz.png]

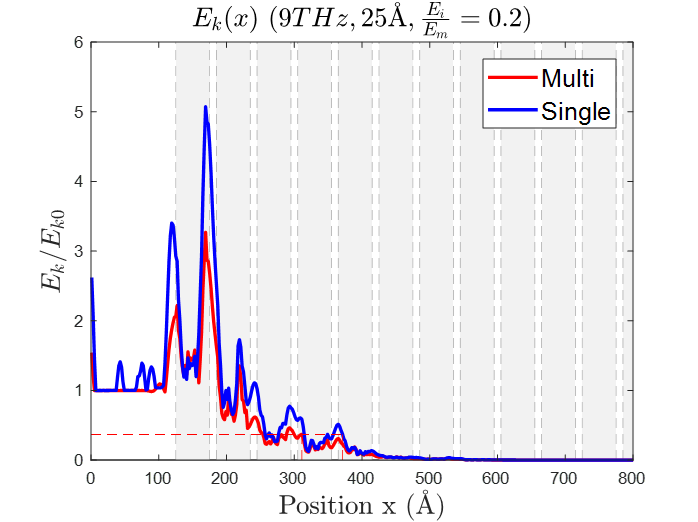

Supplement: Supplementary file 1 [file nanomaterials-09-01471-s001.zip › SupplementaryMaterial/WP_ri=0.2_R=25_9THz.png]
